# Transition prediction in the Ising-model

**DOI:** 10.1371/journal.pone.0259177

**Published:** 2021-11-04

**Authors:** Manfred Füllsack, Daniel Reisinger

**Affiliations:** Institute of Systems Sciences, Innovation and Sustainability Research, University of Graz, Graz, Austria; Helmholtz Centre for Infection Research (HZI), GERMANY

## Abstract

Dynamical systems can be subject to critical transitions where a system’s state abruptly shifts from one stable equilibrium to another. To a certain extent such transitions can be predicted with a set of methods known as early warning signals. These methods are often developed and tested on systems simulated with equation-based approaches that focus on the aggregate dynamics of a system. Many ecological phenomena however seem to necessitate the consideration of a system’s micro-level interactions since only there the actual reasons for sudden state transitions become apparent. Agent-based approaches that simulate systems from the bottom up by explicitly focusing on these micro-level interactions have only rarely been used in such investigations. This study compares the performance of a bifurcation estimation method for predicting state transitions when applied to data from an equation-based and an agent-based version of the Ising-model. The results show that the method can be applied to agent-based models and, despite its greater stochasticity, can provide useful predictions about state changes in complex systems.

## Introduction

Many ecological systems can experience sudden regime shifts where the state of a system abruptly shifts from one stable equilibrium to another. These shifts are usually difficult to predict from the system’s observable states and often involve transitions from preferable to detrimental equilibria. Commonly referred to as critical transitions, they are currently broadly investigated for ways to predict them some time before they occur. Successful methods in this regard are added to a catalogue of methods known as early warning signals (EWSs). While it is important to develop novel methods for this purpose it is equally important to thoroughly test and evaluate these methods’ potential application in a broad range of systems and their model representations. This study sets out to do this with regard to a method for estimating equilibria curves in the vicinity of bifurcations as suggested by a team at the University of Michigan [1–3]. More specifically, this study investigates this method’s performance for predicting state transitions when applied to data simulated by an equation-based (EBM) and a corresponding agent-based model (ABM).

When evaluating novel transition prediction methods, the majority of studies so far focus on analyzing data simulated with EBMs and mostly ignore ABMs with few known exceptions [4]. A core difference between those two modelling paradigms is that EBMs primarily consider the aggregate of a system’s interaction dynamics most commonly represented in the form of coupled differential equations (e.g. for algal bloom and eutrophication in lakes [5–7], for the deterioration of coral reefs [8], for climate change dynamics [9, 10] and for volatility effects in financial markets [11] among others). Therefore, in EBMs, noise as a representation of the system’s fluctuations needs to be “manually” added to the “deterministic skeleton” [12] of the equation. What is more, the addition of noise is not always done with a clear concept of what kind of noise would be appropriate [13]. ABMs, on the other hand, focus on a system’s component level by considering individual agents and their local interactions. These local interactions cause or generate system fluctuations which make stochasticity an endogenous consequence of the modelling approach. It is certainly true that stochasticity of ABMs can also depend on aspects such as the size of the agent population considered, the initial positioning of agents at setup, or the agents’ updating regime. On the consideration, however, that noise has a constructive function in the method at hand ([12] and below), ABMs could prove more appropriate for data generation in this case than EBMs.

The overall objective in this regard is always to generate the most suitable data for the specific evaluation. In the case of assessing the performance of transition prediction methods, noise is of prime importance. While it may be seen as nuisance in other contexts (necessitating computationally expensive evaluations), it is taken to be the actual “creator” of signals in these methods [12] because their core bases on a phenomenon called critical slowing down (CSD). CSD describes a system’s tendency of slowing down recoveries from perturbations when approaching a critical transition [14, 15]. These changes in recovery times thus can signal the onset of a critical transition. In the framework of EWS-analysis, the (small) fluctuations caused by “natural” noise in the dynamics of a system’s variable are seen as ongoing (small) perturbations to the absolute equilibrium of the system, from which it is forced into a sort of permanent recovery activity. Dependent on how far the system is from an equilibrium shift (i.e. a bifurcation), this activity varies in intensity, which can be statistically measured with EWS-methods. In this sense, noise provides the signals that help to understand the state a system is in (see for other constructive aspects of noise also [16]). As mentioned, noise generated by ABMs appears to be more directly integrated, since resulting from component interaction. EBMs, which represent just the clean mathematical “skeleton” of a system’s dynamics, need to be “artificially” equipped with noise in order to provide the required signals. It thus seems counterintuitive that most research on transition predictions focuses on data generated by EBMs. All the more so as the analysis of CSD does not depend on the mathematical representation of a system. It simply requires a way to systematically test systems for their reaction to perturbations, which can be done with either kind of model [13, 17].

However, noise may not be the only reason to consider ABMs as an alternative for investigating possibilities of transition predications. [4] analyze an ABM representing an abrupt transition from low to high unemployment in the course of an economic recession, which is not met by a symmetric decline of unemployment when the economy recovers. The typical hysteresis effect in this model is due to an assumed skill-updating regime of agents driven on the one hand by the state of the economy and on the other hand by the skill-updating regime of an agent’s neighborhood. Visualization reveals that this neighborhood can lock the agents into a behavior which does not correspond to the one that is suggested by the state of the economy. Obviously, a neighborhood negligent in regard to maintaining job chances can determine the skill-updating probability of its members stronger than the actual state of the economy, thus causing delays in employment reinvigoration. This lock-in effect, which creates hysteresis, is not to be observed without a representation of the micro-level interactions in the system and is thus not seen in an EBM.

**Fig 1:**
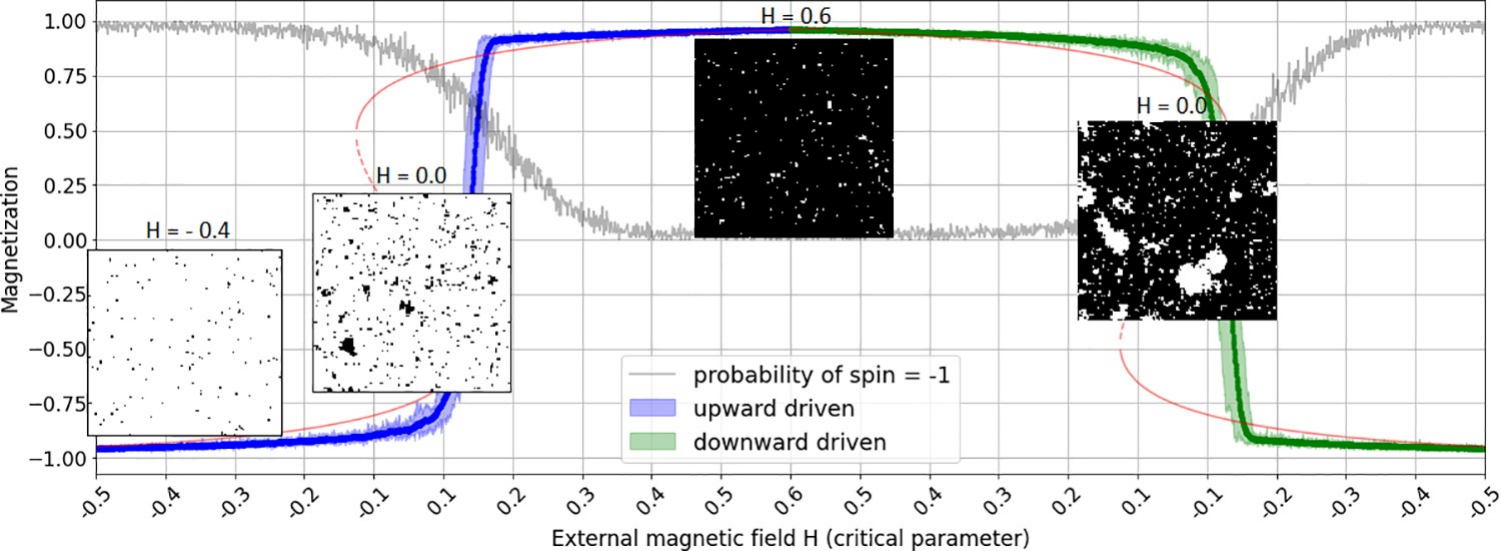
Example simulation of the 2D Ising model. Simulation of the 2D-Ising model with an external magnetic field H taken as critical parameter (see Eq 1), on the x-axis, first increasing (H = -0.5 to 0.6, left half) and then decreasing (H = 0.6 to -0.5, right half). The curve (first blue, then green) shows the overall spin orientation, i.e. the magnetization. Note that its shifts occur at different parameter values in increase and decrease, thus showing hysteresis. The insets visualize snapshots of particle neighborhoods, with insets 2 and 4 taken at the same critical parameter value (H = 0) once in increase and once in decrease, but show distinctly different neighborhood orientations, both though with the characteristic power law distributed cluster sizes. The grey curve indicates the probability of the center patch at (0, 0) being in spin state -1 (white).

Similar may be true for the case of the synchronization study on pistachio masting in the frame of an Ising-model as undertaken by [18]. Fig 1 shows a simulation run from a cellular automaton-based version of this Ising-model with the critical parameter on the x-axis first increasing and then decreasing. The overall spin orientation shifts abruptly at different parameter values in increase and decrease, thus showing hysteresis. [18] interpret the abrupt shifts in the Ising-model as synchronization onsets and muse about possible interpretations for the external field (number of winter-chilling hours for trees, amount of precipitation falling during the spring flowering season) and for the coupling mechanisms (root grafting, trophic interactions with microorganisms etc.). The behavior lock-in, as insinuated in the neighborhood visualizations in Fig 1 (insets 2 and 4), may help to interpret this mechanism. At a minimum, it seems to suggest an information advantage in adjacent agents, e.g. pistachios, possibly from chemical signaling, maybe based on natural root grafting effects [19]. Clearly, the concerted neighborhood behavior–e.g. masting–seems to cause delays in regard to the changes in the external field parameter, revealing a second dynamic which appears to superimpose the first one at least for a short period close to the tipping, thus causing the hysteresis. This second dynamic is not to be seen in an EBM representation of the system. It reveals itself only with regard to local interactions, which can be taken as another argument for considering ABMs as a complementary method for investigating possibilities to anticipate critical transitions in complex systems.

As mentioned, recent studies [18, 20, 21] suggest the Ising-model on ferro-magnetism as suitable for the investigation of regime shifts in ecological systems. Some examples include concerted changes in oscillating ecological populations and the synchronization of masting in trees [18]. We used this system for generating comparable test data. Originally, the Ising-model was suggested in physics to simulate particle behavior in magnetic fields as a sort of cellular automaton [22]. The 2D version of this Ising model is known to exhibit rapid phase transitions between different equilibrium states when a slowly driven parameter passes a critical threshold [23]. In this variant, the system foresees a spatial distribution of particles on a grid and thus considers a causal dependence of spin-states among neighboring particles which, due to their local interaction, generate “natural” fluctuations in the aggregated variable of magnetization. With certain limitations (We formulate carefully here because in this CA-variant too the dynamics of spin changes are co-driven by an equation and not purely rule-based as would be characteristic for ABMs. See for details section 2.) this cellular automaton based Ising-model can be seen as an agent-based model (considering particles as agents) and is therefore referred to as an ABM in this study. However, the Ising model is also commonly considered with a mean-field approximation of interaction in an equation-based variant, i.e. an EBM, which shows similar though not exactly equivalent behavior to the ABM variant.

Since the correspondence of system representations with EBMs and ABMs is still not clear in all its respects, comparisons suggest themselves, especially with regard to their sensitivities in transition prediction. As only few well-established models exist for representing a system in both modeling methods, the suggestion of the Ising model to study regime shifts in ecological systems seems suitable. As said, its EBM as well as its ABM version are widely investigated and the system it represents is known to exhibit different kinds of critical transitions, which can be interpreted in ecological terms. In recent studies, the EBM as well as the ABM-version of the model have been subjected to “classical” EWS-investigations [13, 24]. Here, different to those studies, both versions of the Ising will be subjected to a novel approach for a CSD-based estimation of a system’s equilibria curves around a bifurcation. This approach too bases on CSD but takes a different course as will be explained in section 3.

The rest of this paper is organized as follows: First, the system is introduced as used in this study in its equation-based as well as in its agent-based representation for two of its bifurcation types. Second, the method used for the bifurcation prediction is introduced. Third, results of the investigation are shown. And fourth, gained insights and some limitations of the study are discussed and an outlook on further research is given.

## Ising model

As a simplified model of particle behavior in magnetic metals, the Ising-model on ferromagnetism [22] is known to exhibit rapid transitions between different equilibrium states at certain parameter values. In its original version, the system represents magnetic particles with atomic spins as points on a lattice which can be in one of two states (+1 or −1) dependent on an ambient temperature T and an energy H. Spins are seeking a low energy state causing them to flip abruptly depending on a potential gain in energy which determines a flipping probability. Consequently, as temperature increases, flipping to a higher energy state becomes more likely. However, as the energy to be gained by flipping increases, the likelihood of flipping decreases. Flipping thus depends on the ambient temperature as well as on the state of a particle’s neighbors (in this case a von Neumann neighborhood). With this consideration of spatial interactions and neighborhood influence, the Ising model is often applied to non-physical contexts as well, such as social and economic [25, 26] and lately ecological contexts [18, 20, 21].

**Fig 2:**
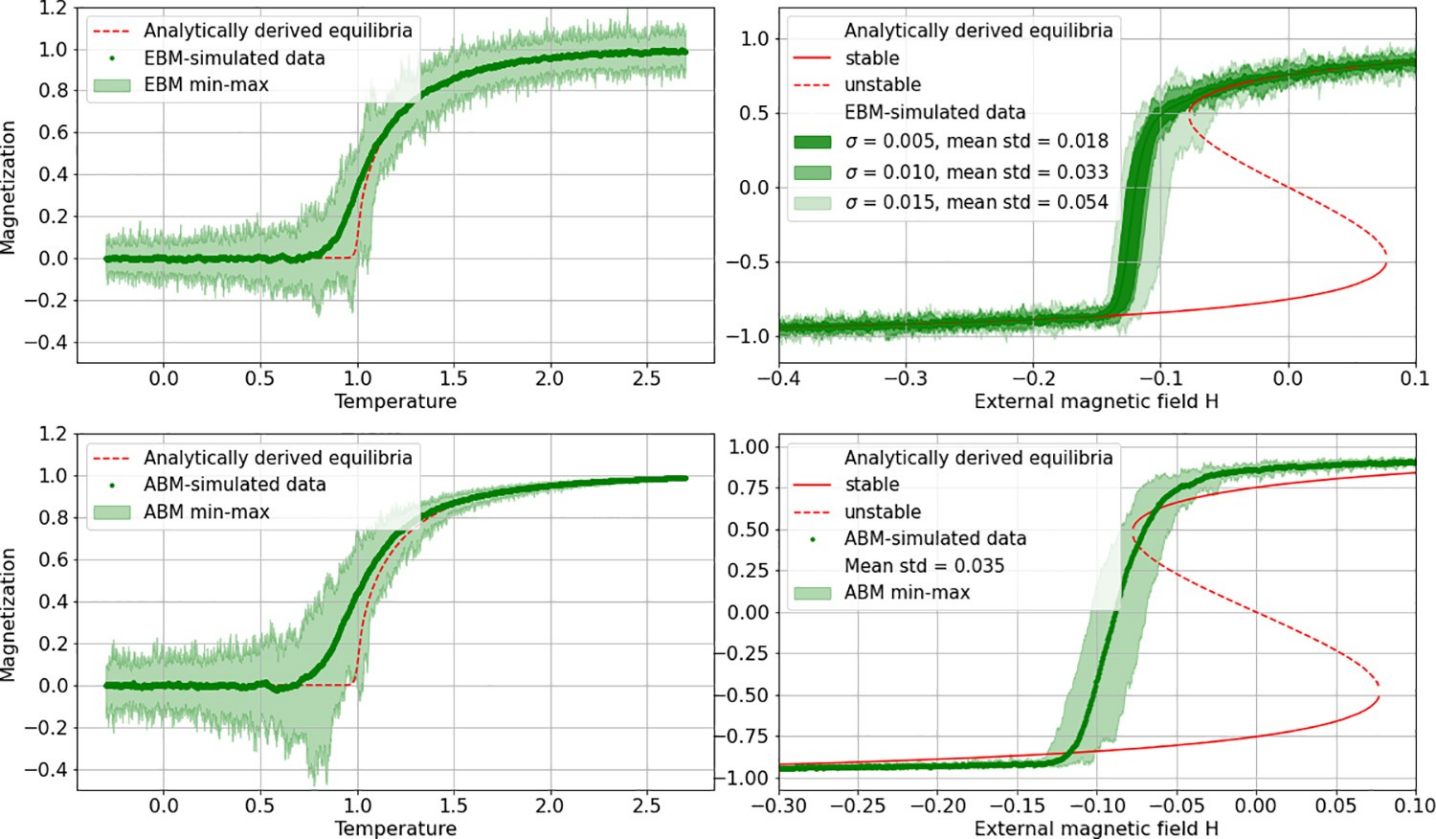
Simulation data of EBM and ABM Ising implementations. Simulation data (min, mean, max from 100 realizations each) and analytically derived equilibria of Ising implementations as EBM (top row) and ABM (bottom row) with pitchfork (left column) and combined saddle node bifurcation (right column). Noise in the case of the EBM realizations has been added according to Eq 1. Note how in the case of the ABM realization the change in noise level distributes like expected primarily around the bifurcations, while in the EBM case noise is more equally distributed (since added) over all simulated data. Additionally, in the EBM saddle-node case the noise-addition has been adjusted (see below for details) so that the signal’s standard deviation (std) best resembles the one of the ABM data. Obviously, a noise level of σ = 0.01 comes close to the one from the ABM realizations. The analytically derived equilibria-curves (red) in the ABM realizations (right column) are generated from the EBM-model and hence approximations. In particular the ABM saddle-node data (bottom right) deviates significantly from these theoretic equilibria.

The system has a well-established equation-based version in form of a mean-field approximation [24, 27], but is also often considered in its 2D lattice-version [23, 28]. Analytically, the system is known to exhibit a (symmetric) supercritical pitchfork bifurcation at a critical temperature value. It may also be locked into a bi-stable region with two stable and one unstable state, thus generating a combined saddle-node bifurcation showing hysteresis with response to an external magnetic field. Both kinds of bifurcations were considered in its EBM as well as in its ABM version (see Table 1 and Fig 2 for an overview).

**Table 1:** Overview of model representations for the pitchfork the and hysteresis system.

	Pitchfork bifurcation	Combined saddle-node bifurcation with hysteresis
**Equation-based version**	Mean-field approximation with varying temperature and no external field influence (H = 0)	Mean-field approximation with varying external field and fixed temperature (T = 2.12)
**Agent-based version**	2D-lattice with varying temperature and no external field influence (H = 0)	2D-lattice with varying external field and fixed temperature (T = 2.12)

### Pitchfork realization

For generating EBM data, the following difference equation was used as a mean-field approximation of the 2D Ising model [24, 27]:

$$M_{t+1}={(-M_{t}+tanh(T∙M}_{t}+H))∙\Delta t+\sigma ∙N$$ (1)

where M is the system’s state or magnetization at a specific point in time, Δt = 0.1 the size of the time-step and T is the temperature and also the critical parameter in the case of simulating the pitchfork bifurcation. H is an external magnetic field, which is fixed at H = 0 in the case of simulating the pitchfork bifurcation. Variability (or noise) was added using a standard normal distribution N, where the distribution’s weight is controlled by the parameter σ. The selection of this addition is critical, but rarely well justified. In this case, we followed [29] in the assumption that most physical systems have a limited amount of available energy per time-step, i.e. a power limitation equivalent to a limit on the variance of a quantity which is best modelled with a normal distribution. A similar modelling procedure is shown in [12].

The corresponding ABM data was generated with a spatial realization of the 2D Ising model, where particles (considered as agents with two spin states) are arranged on a lattice interacting in a von Neumann neighborhood to derive a probability for flipping the spin of a particle through taking the exponential of the ratio of temperature T and negative energy that a particle can gain by flipping. The potential gain in energy is calculated as:

$$E_{diff}=2S(NB-H)$$ (2)

where *S* is the spin of the considered particle, *NB* is the sum of the spins of the particle’s neighbors and *H*H is the external magnetic field, serving as the critical parameter in this case. The particle is then considered to flip its spin

$$\mathrm{if}\;E_{\mathrm{diff}}\leq 0\;\mathrm{or}\;p<\exp(\frac{-E_{\mathrm{diff}}}{T})$$ (3)

where *T* is the temperature and *p* is a random real number between 0 and 1. The mean of the particle spins is taken as the observable aggregated magnetization. The model is actuated using the Metropolis algorithm (adapted from [24, 30, 31]) on a *M* x *M* grid with *M* randomly ∈ {50, 100} and with time series being averaged over 100 realizations each. As said, in case of the pitchfork system temperature *T* is taken as the critical parameter, which when varied across a certain parameter space shifts the system’s magnetization *Mag*, defined as the average spin of all spins on the lattice, mildly abrupt from an unorganized (*Mag* = 0) into an aligned state (here *Mag* = +1) or back.

### Saddle-node realization

To generate the equation-based test data for the saddle-node system, Eq 1 is considered with a varying external field *H*, while fixing the temperature at *T* = 2.12. Here again, variability is added using a standard normal distribution *N*, where the distribution’s weight is controlled by the parameter *σ*.

To generate the agent-based test data for the saddle-node system, an analogous spatial realization was used as in the pitchfork case, with temperature fixed at T = 2.12 and the external field *H* varied linearly between -0.2 and 0.2, causing magnetization to undergo a distinct phase transition. Depending on the direction *H* is varied, a pull or push towards one of the stable states is created. Raising the strength of the field temporarily, can cause the system to shift from its current stable state to the alternative one. However, raising this strength temporarily to a limited value before a bifurcation actually arises, so that the system can recover from the perturbation, allows to derive information about the critical parameter value at which the system is likely to tip.

## Method

**Fig 3:**
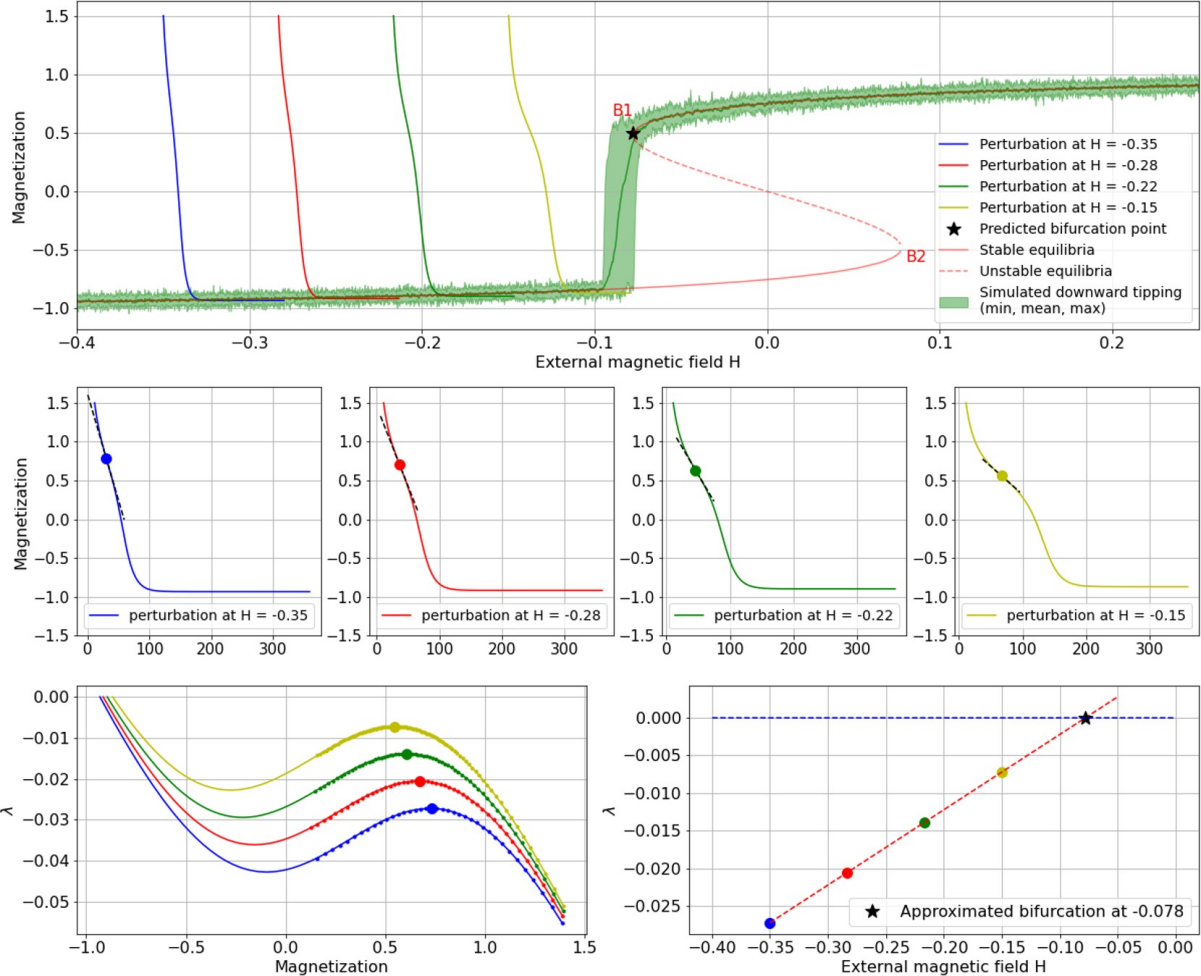
Visualization of transition prediction on EBM-Ising-model (Eq 2) showing hysteresis. Top-row plot) perturbations in relation to bifurcation (B1) with analytically derived equilibria (red); middle-row plots) recovery curves with slope indicated at differentiation maximum; left bottom-row plot) differentiated recovery curves with maxima indicated; right bottom-row plot) linear extrapolation of differentiation maxima against perturbance values. Note that in this realization the amplitude of perturbations is exaggerated for visualization purposes. An amplitude of 1.5 exceeds the depicted aggregate of magnetization, which indicates percentual spin orientation, and is only possible in this EBM-realization.

The method for this derivation is based on the phenomenon of CSD, that is, on the fact that systems driven towards a phase transition exhibit a loss of resilience through prolongations of recovery times when exposed to (small) perturbations. This method can be applied without mathematical representation of the system. It is explained in detail in [3, 4]. Here, it is briefly introduced on the example of EBM generated time series from the saddle-node variant of the Ising model (Eq 1 with non-zero H). The time series are generated at different values of the critical parameter H (blue, red, green and yellow curves in Fig 3 top and middle row plots). Each of these time series is perturbed after a transient time of 100 time-steps by raising the system’s variable beyond the alternate stable equilibrium (Fig 3 top and middle-row plots, where four perturbed time series are shown). Note, however, that the method works with a minimum of two perturbations. The critical parameter values are chosen so that they increase sequentially and that the resilience of the system guarantees a return to the original equilibrium.

As a consequence, CSD can be observed which shows in increasing delays of the recoveries from perturbations. These delays are, as predicted by the underlying theory, caused by the nearby bifurcation point exerting attraction on the system states during recovery. The increasing deflections in the recovery curves can be made evident by calculating the first-order discrete differences of the recovery curves, focusing on the part of their slopes at which the system has not yet returned to its equilibrium and approximating this part with a least square polynomial fit (Fig 3 left bottom plot). Assuming a linear approach to the critical transition and depending on the distinctiveness of the deflection, the maxima of the fitted curves (the bold blue, red, green and yellow points in Fig 3 left bottom plot) can then be considered against the critical parameter values at which the system was perturbed (Fig 3 right bottom plot).

**Fig 4:**
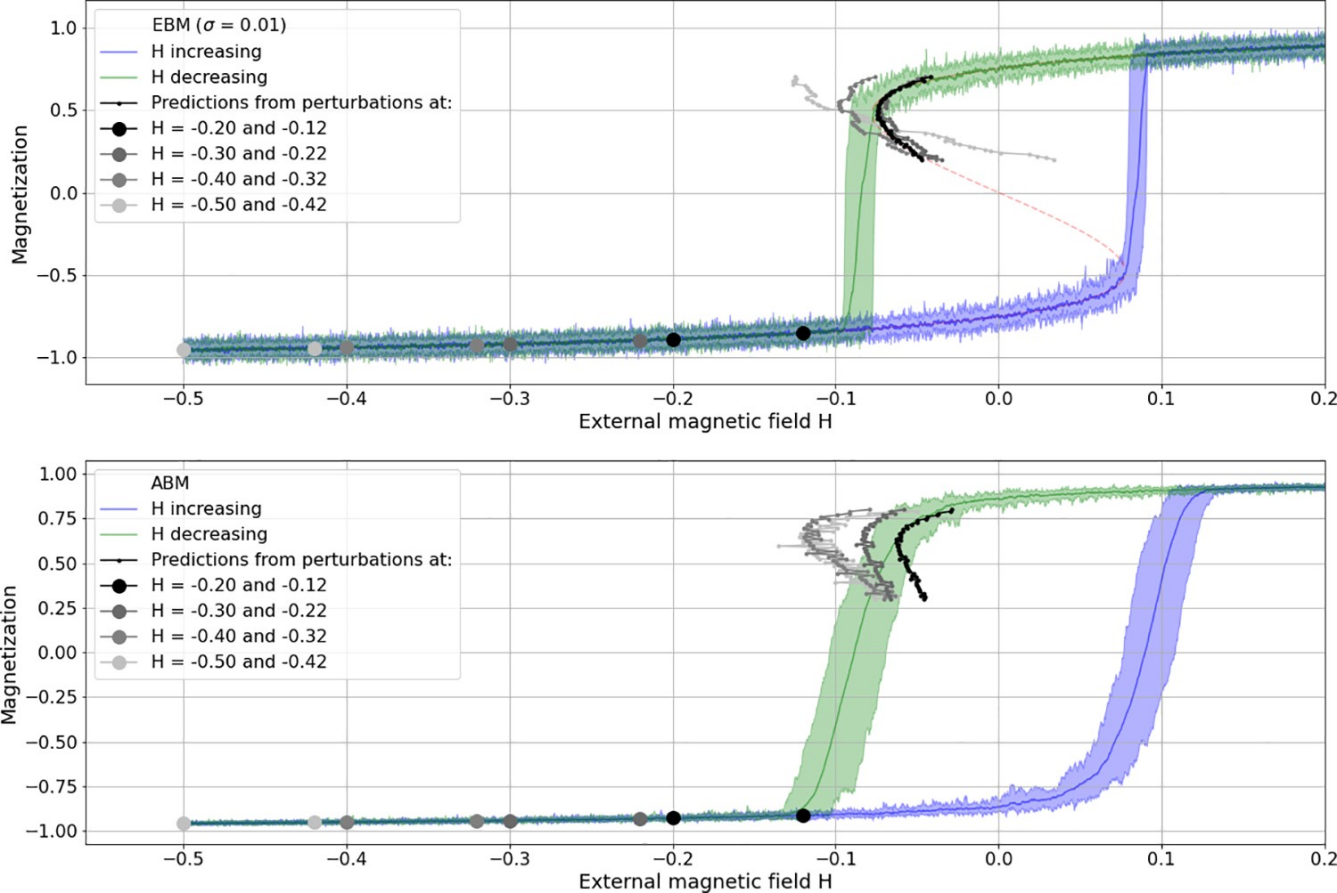
Prediction comparison for the combined saddle-node bifurcation. Comparison for the combined saddle-node bifurcation predictions from perturbations, applied at the lower before-transition range of magnetization on EBM-generated data (top row) and ABM-generated data (bottom row). Light-grey to black dots indicating perturbation parameters, light-grey to black dotted curves indicating corresponding predictions.

**Fig 5:**
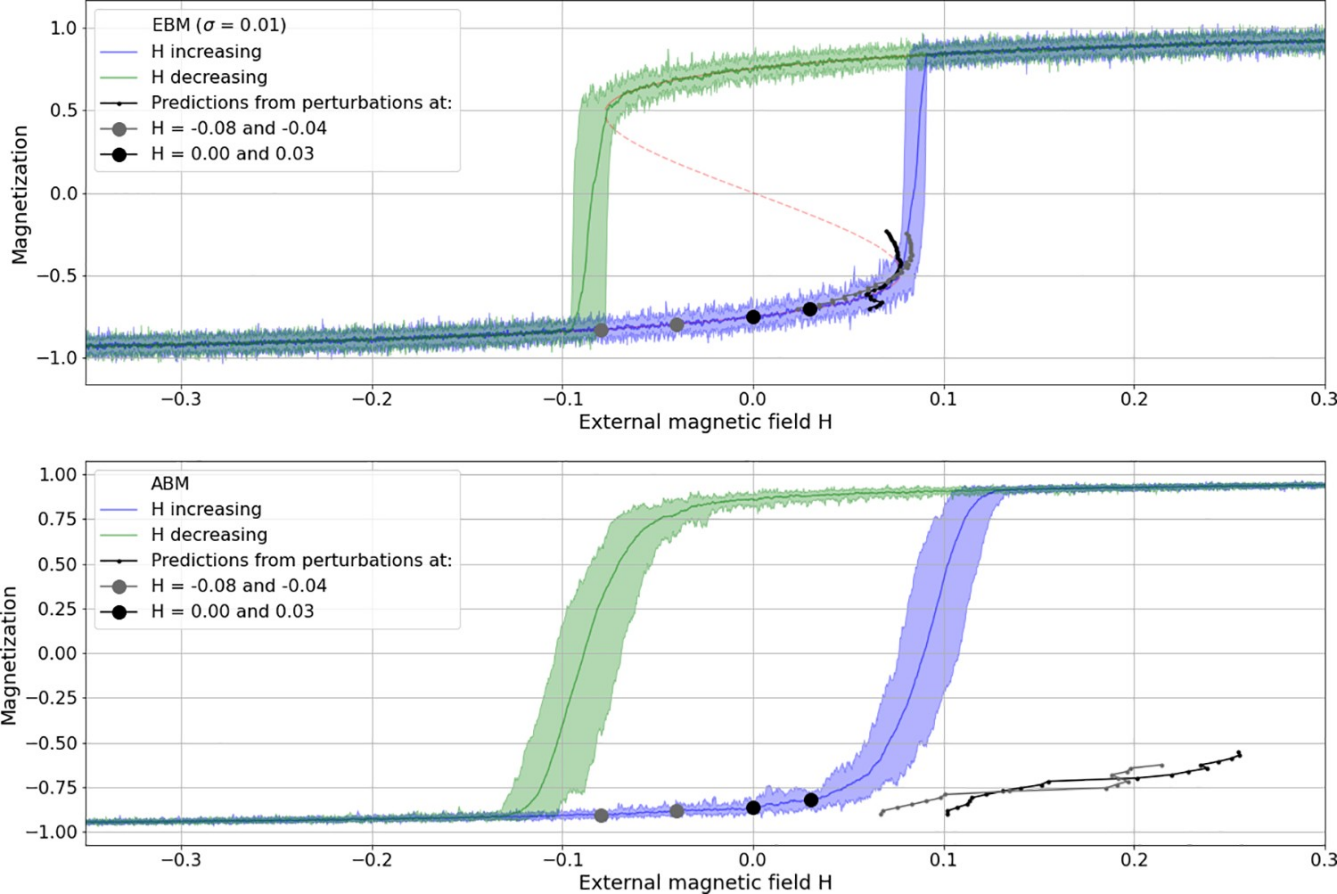
Transition prediction from perturbations to the lower equilibrium branch within the bi-stable region. Light-grey to black dots indicating perturbation parameters, light-grey to black dotted curves indicating corresponding predictions. Perturbation amplitudes kept below the unstable equilibria (red dashed line in top plot) for allowing recovery to the lower equilibria.

Fitting the four points with linear regression and extending the resulting line (dotted red) to the zero-line (dotted blue) indicates the parameter value at which the slope (dotted black in Fig 3 middle-row plots) of a corresponding recovery curve would be horizontal, or in other words, at which no recovery would take place anymore. This point hence indicates the bifurcation point as indicated, in this case at H = -0.078 (Fig 3). Alternatively, a correlated sample of points from the fitted differentiation curves can be obtained, which when treated analogously in a linear extrapolation to the zero line, may provide an approximation of the equilibrium curve around the bifurcation point (see [4] for details, and results in Figs 4, 5 and 7).

Theoretically, an analog procedure should allow to predict the second bifurcation (B2 in Fig 3 top-row plot) in a similar way, with perturbations applied to the lower equilibrium branch within the bi-stable region with amplitudes kept below the unstable equilibrium (see [4]). Despite the need to resort to this procedure to compare the results with “classical” EWS analysis (see below), it did not give promising results, as shown in Fig 5.

## Results

This section shows the results of the application of the described method, with all predictions resulting from averaging over 100 instances of each perturbation in all EBM and ABM cases. In contrast to the explanatory example above, only two disturbances were considered in these cases, which is sufficient for linear extrapolation [3].

### Saddle-node realization

The canonical case for this kind of bifurcation prediction is the combined saddle-node (or fold-) bifurcation which shows hysteresis. Here, the main goal in this regard is to check for the prediction accuracy of applied perturbations in dependence of the distance from the state transition and to compare it between modelling modes. Fig 4 shows four such realizations (light grey to black), with just two perturbations per prediction applied to EBM (top plot) as well as ABM time series (bottom plot).

In both model modes, perturbations were applied by temporarily (i.e. for one time-step) changing the state variable of the system (the aggregated magnetization) to the value of the alternative equilibrium. In the case of the EBM, this meant changing magnetization simply from -1 to 1. In the case of the ABM however, magnetization is an aggregate of the true probability of the spin alignments of the agents. Altering this probability means to have the system develop over some transient time and then disrupt it by changing the alignment probability to the other extreme. In the case of an ordered regime at spin = -1 below the transition range, this meant changing the probability to zero, that is to an unordered state. Consequently, the perturbations do not have the same effect as simply increasing the state variable value as in the case of the EBM. Deviations from the equilibrium are much weaker in the ABM case and hence show less deflection in the recovery curves, i.e. weaker signals. Nevertheless, as can be seen in Fig 4, predictions can be fairly accurate even in the ABM case if perturbations are applied sufficiently close to the expected bifurcation (e.g. H = -0.2 and -0.12). Note that in the EBM case, the noise level of the perturbed time series is adjusted to meet the standard deviation in the ABM time series.

Intuitively, one would want to compare these predictions with signals from what above is tentatively called “classical” EWS-analysis. It should be noted, however, that when applying perturbations to the lower equilibria before the bi-stable region, the predictions obtained therefrom concern the bifurcation of the opposite branch of the equilibrium curve. Hence, the perturbations, as indicated in Fig 4 at the upward bound branch of equilibria (the blue curve), can make predictions for the (upper) bifurcation on the downward bound branch (green curve). In contrast, when the EWS-analysis is concerned with the upward bound (blue) branch, it is expected to signal tipping of the same equilibria branch, that is, the alternative (lower) bifurcation in the hysteresis sigmoid.

As mentioned, the here tested method holds the possibility to apply analogous perturbations to the lower equilibrium branch within the bi-stable region, with amplitudes kept below the unstable equilibria (red dashed line in Fig 5 top plot) so that a recovery to the lower equilibria is possible (see [4]). In the case of the ABM, this meant changing the alignment probability to values not smaller than 0.5 (instead of 0 as in the before-bi-stable-region case). Obviously, with these small disturbances the model did not allow to obtain useful results. Fig 5 shows some of these results: As can be seen, transition predictions are fairly accurate in the case of the EBM realization (top plot). But in the case of ABM generated data (bottom plot) no useful predictions could be derived in spite of extensive parameter sweeps regarding the distance from tipping and the amplitude of perturbations.

**Fig 6:**
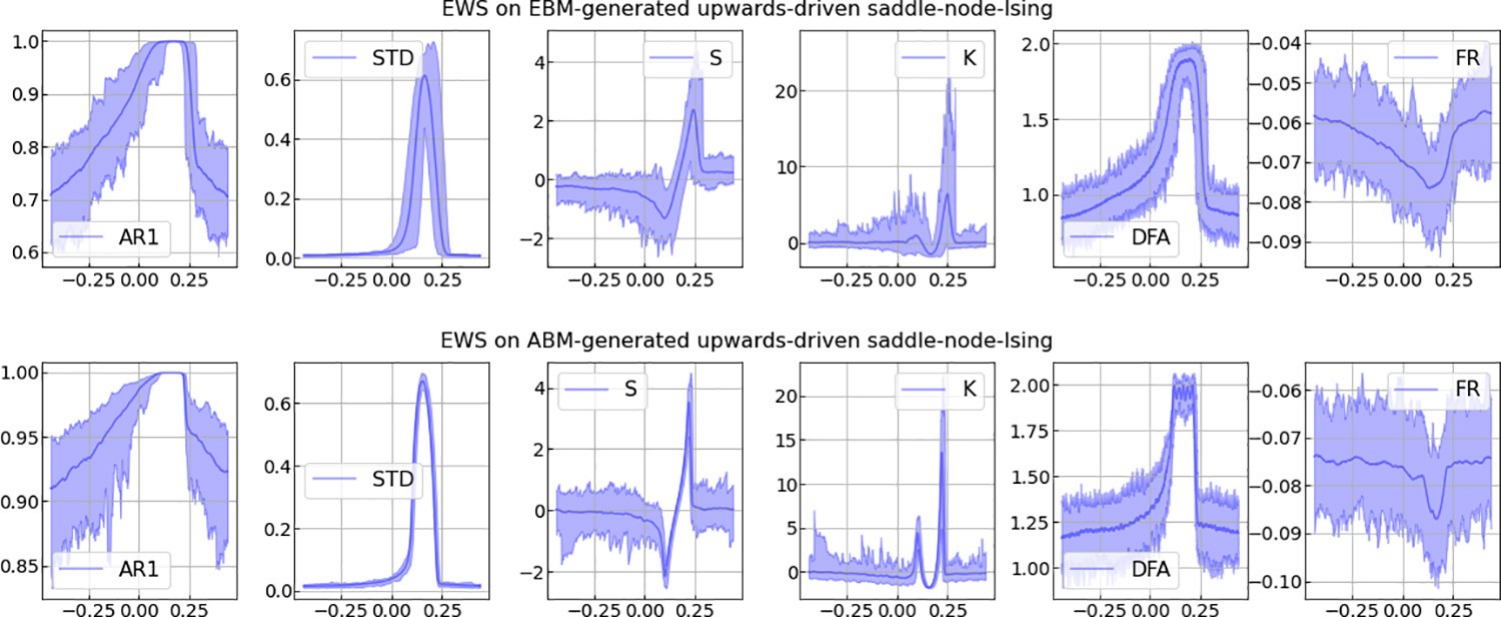
EWS analyses of the saddle-node transition. Results of selected EWS-analyses applied to time series of length 500 generated with rolling windows over the parameter spaces of the upward-driven branch (blue in Fig 5) of the saddle-node transition. Data generated with the EBM- and ABM-models as described in the section: Ising model. Considered EWS-indicators are auto-correlation with lag-1 (AR1), standard deviation (STD), skewness (S), kurtosis (K), detrended fluctuation analysis (DFA) and spectral reddening (FR).

In this case, EWS-analyses may prove as the more reliable method for transition prediction. For comparison, Fig 6 shows results of selected EWS-analyses applied to time series of length 500. The time series were generated with rolling windows over the same parameter spaces as the upward branch (blue in Fig 5) of the hysteresis transition, as generated with the EBM and ABM model described above (for details see [13]).

### Pitchfork realization

**Fig 7:**
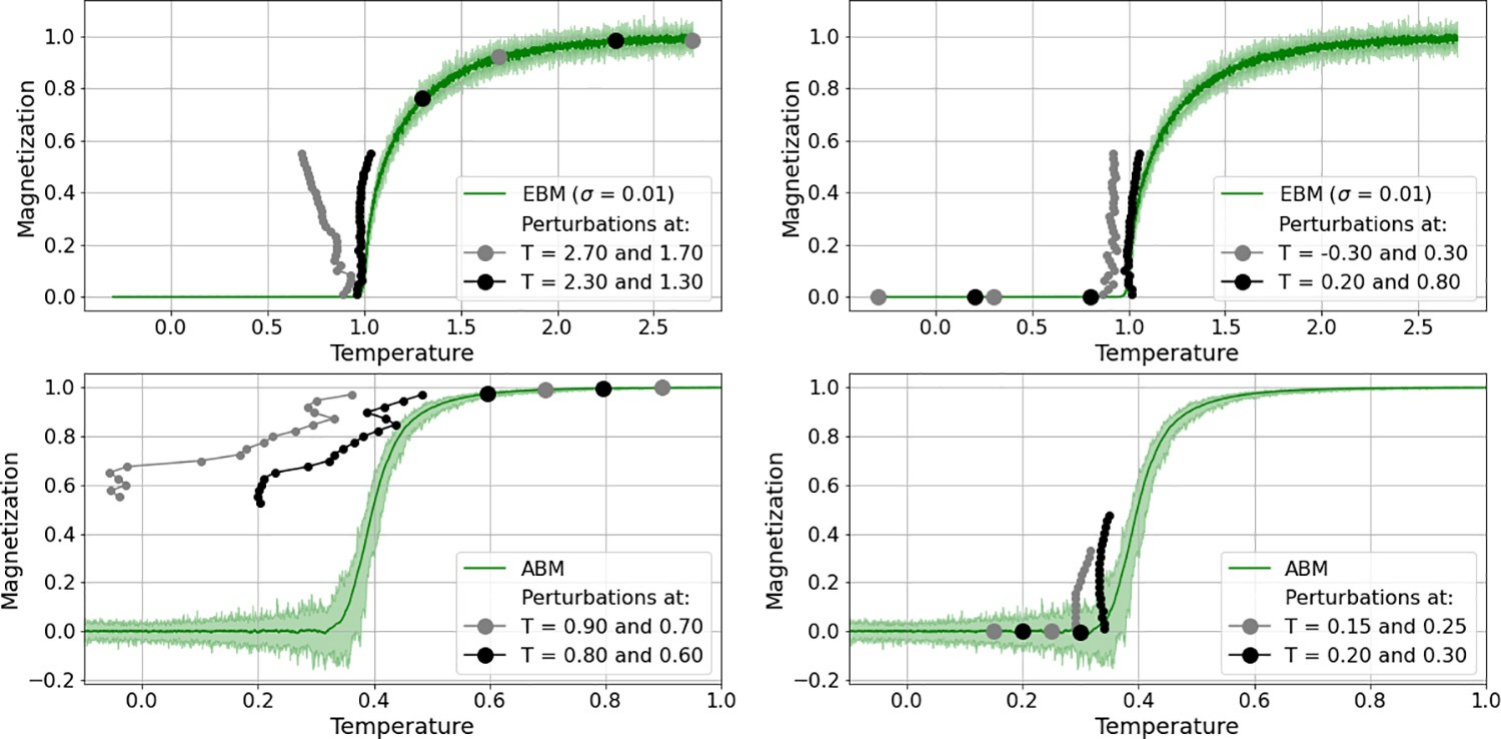
Comparison of pitchfork bifurcation predictions from perturbations. Applied at the above-transition range of temperature (left column) and at the below-transition range of temperature (right column), on EBM-generated data (top row) and ABM-generated data (bottom row).

Despite the rather discouraging results from applying perturbation to the bi-stable region in the hysteresis realization, similar tests were performed for the pitchfork realizations of the considered Ising models. In this case, the critical parameter is the temperature which is known to cause a transition from an unordered state with no spin alignment to an ordered state with spin alignment (either -1 or +1) at a critical temperature value. The tests consider only the upper branch of the pitchfork (spins at +1) and concern a) the direction from which the critical transition is approached and b) the distance of perturbations from the critical transition. Fig 7 shows some of the results: the top row describes the results for EBM-generated data and the bottom row for ABM-generated data. The left column shows bifurcation predictions from perturbations applied at the above-transition range of temperature in different distances from the transition (grey and black dots), and the right column shows predictions from the below-transition range of temperature. In both cases, the distance to the critical transition affects the accuracy of the prediction. Perturbations further away from the CT (grey) produced less accurate predictions.

Again, perturbations were applied by changing the state variable of the system, i.e. the magnetization, for one time-step after some transient time to a value at or close to the alternative equilibrium (to 1 when approaching from the below-transition state and to 0.01 when approaching from the above-transition state). In the case of the ABM, here the same values were applied with the difference that magnetization is an aggregate from the probability of the spin alignment. Altering this probability does not have the same effect as simply raising or lowering the state variable value. Therefore, deviations from the equilibrium are weaker in the ABM case and recovery curves show less deflection. Consequently, perturbations had to be applied much closer to the transition in order to achieve comparable predictions.

**Fig 8:**
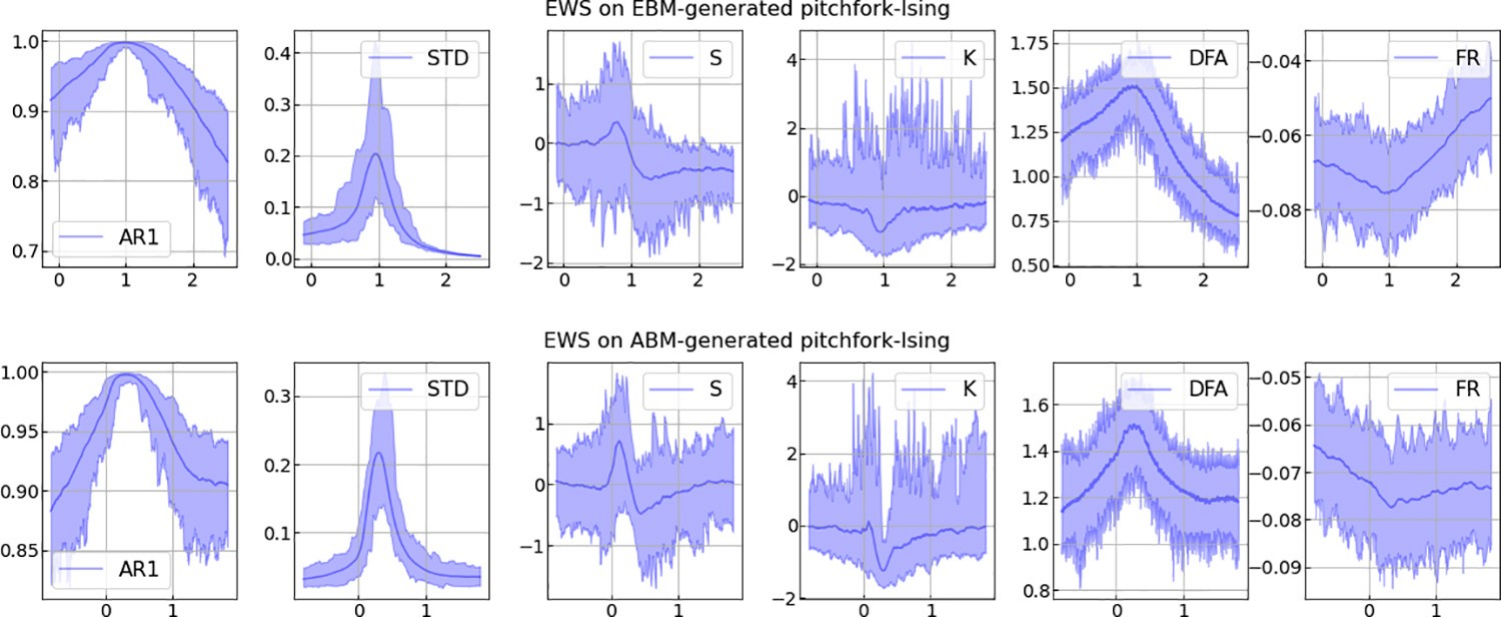
EWS analyses of the pitchfork transition. Results of selected EWS-analyses applied to time series of length 500 generated with rolling windows over the parameter spaces of the pitchfork transition. Data generated with the EBM- and ABM-models as described in the section: Ising model. Considered EWS-indicators are auto-correlation with lag-1 (AR1), standard deviation (STD), skewness (S), kurtosis (K), detrended fluctuation analysis (DFA) and spectral reddening (FR).

For comparison, Fig 8 shows the results of selected EWS-analyses applied to time series of length 500 generated with rolling windows over the same parameter spaces as the pitchfork transition (for details see [13]).

## Discussion

The experiments show that the prediction of critical transitions with the bifurcation estimation method as described in the method section is possible on ABM-generated data and may, with certain limitations, provide useful results. The limitations primarily concern stochasticity which can obfuscate predictions severely. However, this is an endogenous effect in ABMs and thus may provide an estimate of the difficulties the method may encounter when applied to real-world data. Implementing perturbations by way of altering the probability of certain micro-level behavior may well conform to what needs to be done in experiments for predicting transitions in ecological systems. In this respect, the simulation with EBM methods rather hides the possibilities for interfering with real systems and can therefore trigger unjustified expectations regarding the potential of transition predictions.

An interesting aspect, which is of particular importance in the case of systems that undergo the combined saddle-node transitions with hysteresis, seems to be the explicit consideration of spatially distributed local interactions in ABMs. What is called the “second dynamic” of neighborhood interaction in the agent-based Ising-model, causing local behavior lock-ins and thus delayed reactions to the driving parameter, may remain hidden in EBM representations. While it is sometimes found that the resilience of an equilibrium depends on the negative feedbacks that stabilize that state, a detailed analysis of the interactions that cause these feedbacks is rarely provided. This is especially true for abbreviated representations in EBMs which mostly draw their attention to the macro level of a system. The results presented here—in particular some tests with EWS indicators for the spin probability of selected particles, as shown in Fig 1 (gray), which will be detailed in another article—give rise to the assumption that a more detailed analysis of the local interactions in systems, possibly also via network representations, can provide deeper insights into the causes and dynamics of critical transitions and thus further the exploration of transition predictions. In this regard, ABM-representations of ecological systems seem to provide a promising alternative to the prevailing method of investigating system dynamics by way of equation-based methods.
